# Musculoskeletal ultrasound workshops in postgraduate physician training: a pre- and post-workshop survey of 156 participants

**DOI:** 10.1186/s12909-019-1769-6

**Published:** 2019-09-23

**Authors:** Wei-Ting Wu, Ke-Vin Chang, Der-Sheng Han, Levent Özçakar

**Affiliations:** 1 Department of Physical Medicine and Rehabilitation, National Taiwan University Hospital, Bei-Hu Branch and National Taiwan University College of Medicine, Taipei, Taiwan; 2 Department of Physical and Rehabilitation Medicine, Hacettepe University Medical School, Ankara, Turkey

**Keywords:** Ultrasonography, Rehabilitation, Course, Education

## Abstract

**Background:**

Use of ultrasonography has revolutionized diagnosis of musculoskeletal disorders. Until now, few studies have investigated usefulness of a short-period workshop for musculoskeletal ultrasound (MSKUS) education. In this research, we attempted to explore (1) whether the physicians felt it useful to attend this type of courses for improving knowledge of sonoanatomy and scanning skills, (2) if the attendees’ perceived confidence in musculoskeletal diagnoses by using ultrasound increased following the program and (3) whether differences existed in perceived usefulness and confidence regrading different sessions of the course.

**Methods:**

The target participants of the courses were postgraduate physicians without limitation of their specialties. The attendees’ responses to questionnaires before and after the course were reviewed. The workshop contained didactic and practical sessions on 6 major joints in accordance with the scanning protocols of EURO-MUSCULUS/USPRM. The course usefulness and perceived confidence in MSKUS examination were evaluated using a 5-point Likert scale. Data relevant to participants’ pre-workshop confidence levels were also analyzed. If any participant attended the course for more than 1 time, only their first survey was used for analysis.

**Results:**

The study included 156 participants. The average rating for the course usefulness ranged between 4 (useful) to 5 (very useful). There was no difference in perceived usefulness between the didactic and hands-on practical sessions. Participants’ perceived confidence significantly increased after the workshop but appeared to be lowest for evaluation on the hip joint. Previous experience in performing MSKUS (in years) was consistently associated with the level of pre-workshop confidence.

**Conclusion:**

A short period ultrasound workshop might be useful regarding making musculoskeletal diagnoses by using ultrasound based on an increase in post-workshop confidence in MSKUS examinations. The perceived confidence of hip scanning was lower than that of other joints after the course, indicating inadequacy of education in hip sonoanatomy and intensity of hand-on practice in the present program. An increase in the faculty-to-student ratio or length of practice in the hip section should be implemented in the future course.

**Electronic supplementary material:**

The online version of this article (10.1186/s12909-019-1769-6) contains supplementary material, which is available to authorized users.

## Introduction

In recent years, the use of ultrasonography has revolutionized the diagnosis and management of musculoskeletal disorders for physicians [1]. It has gained prominence for its zero-radiation, portability, multi-modal assessment, and real-time guidance of interventions. Like cardiac, renal, breast, and gynecological sonography, musculoskeletal ultrasonography (MSKUS) comprises dynamic evaluations and/or assessments of different structures (the ligaments, tendons, muscles, vessels and nerves [2–8]). In the musculoskeletal field, the limitations of ultrasound imaging techniques include exaggerated attenuation in deeper structures and obese patients, existence of numerous normal variants and a limited window for visualizing a target behind the bone. Nevertheless, the aforementioned disadvantages can be substantially overcome by improving physicians’ knowledge of regional sonoanatomy and skills of adjusting machine settings and investigating challenging structures. Another issue is that ultrasonography has also long been criticized for being operator dependent [1]. Hence, a standardized scanning protocol is also paramount in MSKUS education.

A structured curriculum comprising didactic and practical sessions has been applied for ultrasound education in many specialties. Bernard et al. found that a multimodal educational approach significantly improved the levels of confidence of medical students in scanning the head and neck regions, and that instructor demonstrations were rated as the most crucial component of the program [9]. Recently, point-of-care ultrasound (POCUS) has been increasingly used at patients’ bedside for diagnostic and therapeutic purposes [10, 11] . POCUS also enables emergency or primary care physicians to quickly recognize musculoskeletal and soft tissue disorders and to provide better and safer cares [12]. Kelm et al. revealed that a longitudinal curriculum for POCUS enhanced knowledge retention and the ability to recognize pathological findings for internal medicine residents [13]. Rempell et al. also found that training of POCUS could be successfully administered by integration of didactics and small group hands-on teaching sessions [14]. Therefore, based on the available literature, an organized course integrating multiple components, such as lectures of ultrasound physics and sonoanatomy, along with tutor demonstrations and practice on volunteers, appeared to be the most efficient method of ultrasound education. Starting from 2015, we developed a two-day MSKUS course incorporating the didactic and hand-on practice sessions based on 6 different joints. However, we were not aware whether the short-term course was as helpful as the aforementioned ultrasound curriculum.

Therefore, through reviewing the pre- and post-workshop questionnaires of the attendees, the present research attempted to explore (1) whether the physicians felt it useful to attend a short-period ultrasound workshop for improving knowledge of sonoanatomy and scanning skills, (2) if the attendees’ perceived confidence in musculoskeletal diagnoses by using ultrasound improved following the program and (3) whether differences existed in perceived usefulness and confidence regrading different parts of the course.

## Materials and methods

### Participants

The present study employed a retrospective cross-sectional design. The target attendees of the 2-day course were postgraduate physicians. Most of the attendees were physiatrists. Other participants included orthopedic surgeons, family physicians, emergency medicine specialists, neurologists, anesthesiologists, radiologists, rheumatologists, pediatricians, neurosurgeons, and etc. The workshop has been held annually since 2015 by our department of physical medicine and rehabilitation, in collaboration with the medical ultrasound society. Registration for the workshop was open to physicians from all specialties. The study was approved by the institution’s review board of National Taiwan University Hospital (IRB NO. 201707033 RINB), and the need for informed consent was waived due to the retrospective nature of the research. The data were processed and analyzed anonymously.

### Course arrangement

**Fig. 1:**
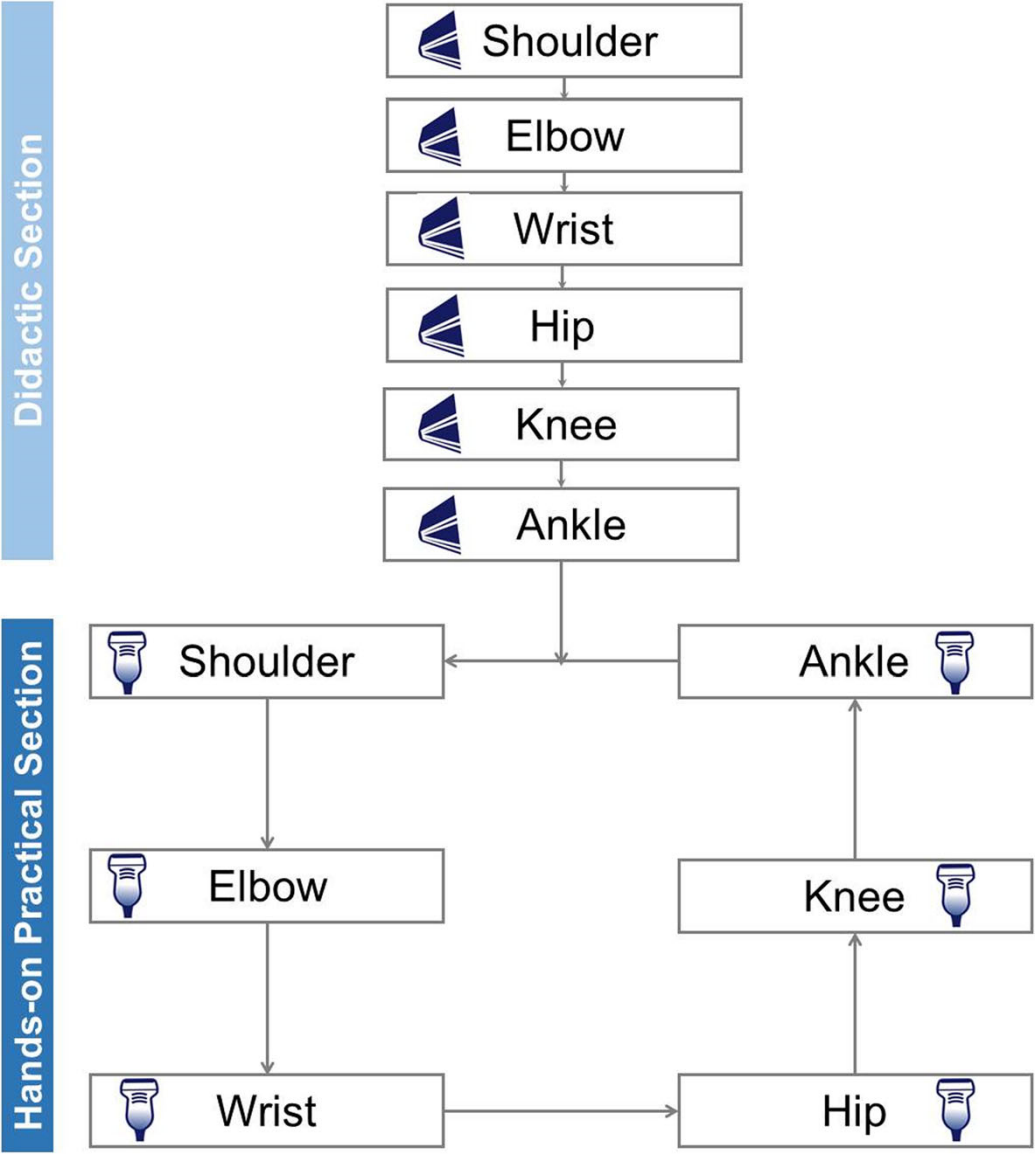
A flow chart showing the arrangement of the didactic and hands-on practical sessions during the workshop

**Fig. 2:**
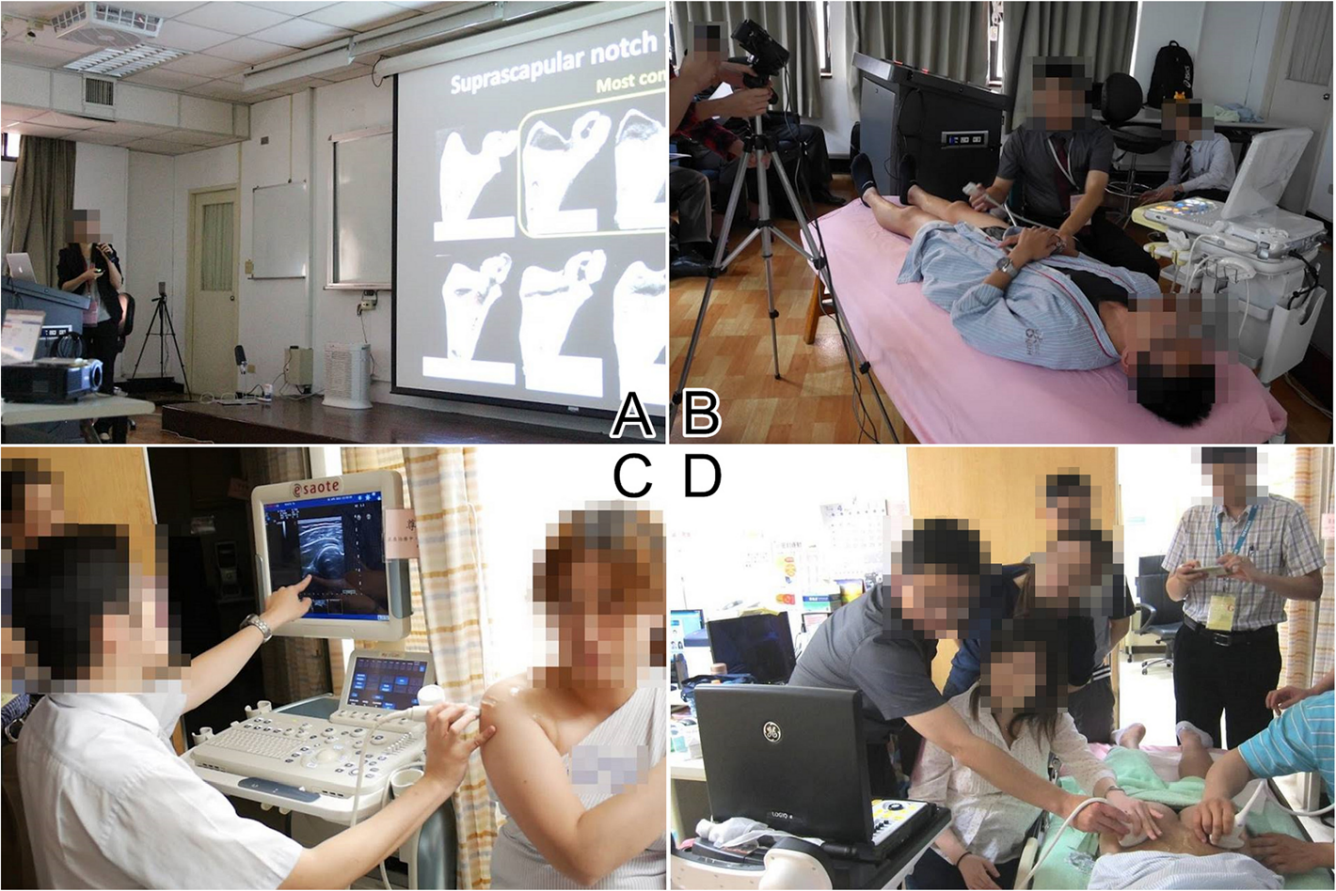
The course was divided into two parts. The didactic section consisted of (**a**) lectures based on six major joints and (**b**) standardization of scanning skills by using projections of ultrasound images and hand gestures on the screen in the auditorium room. The practical section comprised (**c**) demonstration of the sonoanatomy by the instructor and (**d**) scanning on the volunteer model by the attendee under the instructor’s assistance

The workshop consisted of two parts: first, the didactic sessions elaborating fundamental knowledge were given, and then, relevant hands-on sessions focusing on scanning skills were conducted. Both parts included 6 sessions/stations arranged according to 6 joints (i.e., the shoulder, elbow, wrist, hip, knee, and ankle joints) (Figs. 1 and 2). Each station contained a high-end ultrasound machine, a volunteer model, one instructor, and a maximum of 8 participants (for the optimum amount of practice).

During the didactic part, ultrasound knobology, regional anatomy, standard postures during the examination, transducer placement, and ultrasonography findings of normal and abnormal target joints were addressed. The following structures were covered for each joint and scanned in accordance to the scanning protocols of EURO-MUSCULUS/USPRM: (1) for the shoulder: the biceps tendon, subscapularis tendon, acromioclavicular joint, supraspinatus tendon, infraspinatus tendon, and glenohumeral joint [15]; (2) for the elbow: the common extensor tendon, radial collateral ligament, distal biceps tendon, common flexor tendon, ulnar collateral ligament, and triceps tendon [16]; (3) for the wrist: 6 dorsal extensor compartments, triangular fibrocartilage complex, and carpal tunnel [17]; (4) for the hip: the iliopsoas tendon, rectus femoris tendon, anterior hip recess, adductor and gluteus muscle groups, and piriformis muscle [18]; (5) for the knee: the suprapatellar pouch, quadriceps tendon, patellar tendon, medial collateral ligament, iliotibial band, lateral collateral ligament, and posterior cruciate ligament [19]; and (6) for the ankle: the anterior ankle pouch, anterior inferior tibiofibular ligament, anterior talofibular ligament, calcaneofibular ligament, peroneus tendon, deltoid ligament, Achilles tendon, and plantar fascia [20].

In the hands-on sessions, the instructors demonstrated the procedure for scanning the pertinent joint and the participants then practiced under the supervision of the instructors (Figs. 1 and 2).

### Questionnaires

The questionnaires were designed to examine whether the course was helpful in making a musculoskeletal diagnosis based on different joints. The attendees were invited to respond to the questionnaires as a feedback to the course for future improvement. Prior to attending the lectures, all participants filled out a pre-workshop survey (Additional file 1) documenting their age, sex, work experience, specialty, MSKUS experience, access to an ultrasound machine, and previous attendance to other ultrasound training programs. Their perceived confidence in MSKUS examination before the workshop was also evaluated for the 6 major joints by using a 5-point Likert scale, with 1 being the least confident and 5 being the most confident.

At the end of the workshop, the attendees filled out another survey (Additional file 2) evaluating their perceived usefulness of the course and perceived confidence in MSKUS scanning based on 6 different joints. Regarding course usefulness, a 5-point Likert scale was used, with 1 being the least useful and 5 being the most useful. They were also asked to choose their topic(s) of interest for future ultrasound courses.

### Statistical analysis

The distribution of continuous/interval variables was expressed by mean and standard deviation values, and categorical variables were expressed by numbers and percentage values. The items rated using a Likert scale were mainly analyzed as interval data, but the distribution of each response was also given. Comparison of means among multiple groups was achieved by analysis of variance (ANOVA) and Bonferroni adjustments for the post-hoc tests. Chi-square test was employed to compare proportions between two or more groups. A paired t-test was used to compare the usefulness between the didactic and hands-on practical parts and the perceived confidence between the pre- and post-workshop evaluations. Factors related to pre-workshop perceived confidence levels were analyzed using a multiple linear regression model. All analyses were performed using Stata version 11 (Stata Corp LLC, College Station, TX, USA), and the level of statistical significance was set at *p* < 0.05.

## Results

### Participants

Three courses had been held annually since 2015, with a total of 186 attendees. If any participant attended more than 1 workshop, only their first survey was used for analysis. After excluding 22 surveys with incomplete responses and 8 surveys of repeated attendance, 156 sets were included in the study.

The mean age of the enrolled participants was 35.9 years (standard deviation: 8.4 years), and females accounted for 28.2% (*n* = 44) of the study population. Regarding their workplaces, 61.6% (*n* = 96) of the attendees came from teaching hospitals, 19.2% (*n* = 30) from local hospitals, and 19.2% (*n* = 30) from private clinics. Their average number of working years was 8.7 (standard deviation: 9.2 years). In terms of specialty, physiatrists accounted for 55.1% of the participants (*n* = 86); other attendees included orthopedic surgeons (*n* = 30), family physicians (*n* = 8), emergency medicine specialists (*n* = 7), neurologists (*n* = 6), anesthesiologists (*n* = 6), radiologists (*n* = 5), rheumatologists (*n* = 2), pediatrician (*n* = 1), and neurosurgeon (*n* = 1), as well as several health practitioners who did not specify their specialties (*n* = 4). Among the analyzed surveys, 116 reported having experiences of performing MSKUS examinations earlier, and the mean duration of using it in clinical practice was 2.3 years (standard deviation: 3.8 years). Although 108 participants (69.2%) claimed they had good access to an ultrasound machine, 136 attendees (87.1%) had never attended a MSKUS workshop before.

### Perceived usefulness of the course

When different joints were compared, perceived usefulness was found to be similar for both the didactic and the hands-on sessions. Additionally, the two types of sessions were again found to be similar when each joint was compared separately (Table 1). The average rating for the course usefulness ranged between 4 (useful) to 5 (very useful). The distribution of participants’ responses is given in Table 2.

**Table 1:** Confidence levels and course usefulness of the participants for different joints

	Shoulder	Elbow	Wrist	Hip	Knee	Ankle	*P* value (comparison among joints)
Perceived confidence							
Pre-workshop evaluation	3.03 ± 0.90*^abcd^	2.35 ± 0.96*^aef^	2.41 ± 0.97*^bgj^	2.07 ± 1.02*^cfghi^	2.83 ± 1.03*^ehj^	2.53 ± 0.99*^di^	0.007
Post-workshop evaluation	3.98 ± 0.67*^abc^	3.65 ± 0.80*^aef^	3.71 ± 0.79*^bg^	3.32 ± 0.85*^cfghi^	3.95 ± 0.66*^eh^	3.77 ± 0.73*^i^	< 0.001
Course usefulness							
Lecture	4.51 ± 0.58	4.58 ± 0.57	4.54 ± 0.65	4.45 ± 0.62	4.52 ± 0.59	4.50 ± 0.66	0.33
Hands-on practice	4.57 ± 0.59	4.62 ± 0.57	4.60 ± 0.59	4.47 ± 0.64	4.59 ± 0.57	4.55 ± 0.69	0.32

* significant difference between the pre-workshop and post-workshop evaluations

Post-Hoc analysis:

^a^ significant difference between shoulder and elbow;

^b^ significant difference between shoulder and wrist;

^c^ significant difference between shoulder and hip;

^d^ significant difference between shoulder and ankle;

^e^ significant difference between elbow and knee;

^f^ significant difference between elbow and hip;

^g^ significant difference between wrist and hip;

^h^ significant difference between hip and knee;

^i^ significant difference between hip and ankle;

^j^ significant difference between knee and wrist

**Table 2:** Number of participants for each item pertaining to different joints

	Shoulder	Elbow	Wrist	Hip	Knee	Ankle	*P* value (among-joint comparison)
Pre-workshop perceived confidence							
Not at all	7	30	31	51	18	25	
Very little	36	60	49	62	37	51	
Some	62	50	59	28	60	56	
Confident	47	12	14	10	34	20	
Very Confident	4	4	3	5	7	4	< 0.001
Post-workshop perceived confidence							
Not at all	0	0	1	1	0	0	
Very little	1	9	7	20	0	4	
Some	34	60	50	79	38	51	
Confident	88	63	75	39	87	77	
Very Confident	33	24	23	17	31	24	< 0.001
Usefulness of the lecture part							
Not at all	1	1	1	1	1	1	
Very little	0	0	2	0	0	1	
Some	1	1	2	5	2	6	
Useful	69	59	57	71	66	59	
Very Useful	85	95	94	79	87	89	0.469
Usefulness of the hand-on practical part							
Not at all	1	1	1	1	1	2	
Very little	0	0	0	0	0	1	
Some	2	1	3	7	1	3	
Useful	59	53	51	64	57	52	
Very Useful	94	101	101	84	97	98	0.534

When the participants’ choices for future courses were analyzed, the distribution of topics was as follows: interventional ultrasonography (71.8%, *n* = 112), peripheral nerve imaging (41.6%, *n* = 65), and ultrasonographic imaging of the spine (36.5%, *n* = 57). The proportions were significantly different (*p* < 0.001).

### Perceived confidence for MSKUS examination

Compared with the pre-workshop evaluation, there was a significant improvement in perceived confidence following the curriculum. Participants felt most confident regarding shoulder joint scanning both at pre- and post-workshop assessments. The pre-workshop confidence levels were similar for the elbow, wrist, knee, and ankle joints and the lowest for the hip (Table 1). After the workshop, the attendees still felt the least confident regarding performing a hip joint evaluation, and reported similar levels of confidence for the remaining joints, with significant differences only between the elbow and knee joints. The distribution of participants’ responses is presented in Table 2. There were significant differences among the proportions of each item response for different joints in both the pre- and post-workshop evaluations.

When the pre-workshop perceived confidence was treated as the dependent variable, years of experience in performing MSKUS (Additional file 1) were found to be significantly positively associated with the confidence for all the joints. Additionally, older participant age was observed to be associated significantly with lower perceived confidence for shoulder scanning (Table 3).

**Table 3:** Correlation coefficients among pre-workshop confidence levels and *characteristics of the participants*

	Shoulder	Elbow	Wrist	Hip	Knee	Ankle
Age (year)	−0.03*	0.01	0.00	0.02	−0.02	−0.01
Male gender	0.08	0.12	0.31	−0.05	0.21	0.16
Work experience (year)	0.01	− 0.00	0.00	−0.01	0.00	0.01
Physiatrist	0.28	0.21	0.08	−0.26	0.28	0.22
Experience of musculoskeletal ultrasound (year)	0.07*	0.06*	0.06*	0.06*	0.08*	0.07*
Accessible ultrasound machines	0.22	0.13	0.15	0.17	0.05	0.15
Attendance of other ultrasound training programs	0.13	0.26	0.25	0.01	018	0.08

* p < 0.05

## Discussion

The present study showed that a short-term musculoskeletal ultrasound course appeared useful for postgraduate physicians regarding employing ultrasound to make musculoskeletal diagnoses. The perceived usefulness was not different between the didactic and hand-on practice sessions. However, even after the course, the participants still felt less confident in performing the examination for the hip joint. Of note, previous MSKUS experience seemed to be consistently associated with the level of pre-workshop confidence of the subjects.

In terms of the comparison of usefulness between the didactic and hands-on sessions, we were surprised to find no significant differences. Since mastering the examination skills is more easily achieved by real-time instruction than by slide presentation, we thought that the hands-on practice programs would be rated as more useful than the didactic part. A plausible explanation for this finding could be that in our workshop, the theoretical and hands-on sessions incorporated fundamental sonoanatomy and clinical applications of MSKUS, which made the attendee feel as useful as the hands-on practice session. During the development of the questionnaires, the perceived confidence in MSKUS examination on 6 different joints was incorporated in the pre- and post-workshop evaluation. The perceived confidence, was used as a proxy of course effectiveness for the attendees to apply what they learned on clinical practice. The attendees might feel the program to be useful in improving their knowledge but of little help in dealing with real patients. In order to check if there was an inconsistency between both aforementioned components, the perceived confidence was recorded and also analyzed.

Our finding of improved perceived confidence after the workshop is consistent with that of similar ultrasound curricula [21–23], demonstrating the effectiveness of a short-term course for facilitation of MSKUS examination. Furthermore, since our workshop covered 6 major joints, we were able to analyze whether participants’ perceived confidence varied with respect to scanning different regions. An important finding was that the hip joint was ranked as the region for scanning that attendees were least confident about, whereas the majority felt most confident regarding scanning the shoulder joint. In the standardized training program of MSKUS, the shoulder is usually the first joint to be taught. When physicians start to apply imaging modalities in clinical practice, they find it useful because of the high prevalence of shoulder disorders [24, 25]. Further, the main structures imaged during shoulder examination, such as rotator cuff tendons, are superficial and easily scanned with ultrasonography. Therefore, our finding of high confidence for scanning the shoulder joints seems to be reasonable in this sense.

By contrast, the majority of the participants felt least confident about performing hip joint examination. We speculated at least 2 reasons for this finding. First, most of the main structures in the hip are deeply situated. While this makes physicians less familiarized with the local structures (and their physical examination when compared with other superficial joints), it might sometimes require the use of a curvilinear transducer, for which musculoskeletal specialists are typically less experienced [26]. Second, the scanning skills for some parts of the hip territory (e.g. the piriformis muscle and sacroiliac joint) is more often taught in the curriculum for axial skeleton scanning, and is not routinely discussed in the MSKUS training programs. In this regard, comprehensive instruction and intensive practice appear to be paramount for improving competence in hip ultrasonography.

Our study revealed that the only factor that was consistently associated with the pre-workshop perceived confidence level was previous MSKUS experience, reflecting the number of years that participants had used MSKUS in their clinical practice. We believe that this observation also strengthens the point that the learning curve of MSKUS is never an abrupt rise, and that time spent in practice plays a vital role for the maturation of skills. Another interesting finding was the negative association between the confidence level and participants’ age for shoulder imaging. Since MSKUS had been included in the physiatry resident training programs in our country for approximately 10 years [27, 28], most young participants were aware of basic scanning skills before the course, especially for the shoulder joint, which is usually taught first. In contrast, some older participants tended to score their confidence level low even for the shoulder joint, possibly due to the lack of organized ultrasound education during their residency years.

The strength of our study was that we evaluated the perceived course usefulness and post-learning confidence based on different body regions, which had rarely been reported in previous literature. Our analysis helped identification of a gap regarding the current course arrangement. In the present program, the faculty-to-student ratio and length of hands-on practice were the same at each session. However, our data revealed inadequacy of education in hip sonoanatomy and intensity of hands-on practice. Therefore, we suggested increasing the number of instructors and duration of practice in the hip session in the next workshop. Concerning future courses, interventional ultrasonography seemed to be the most favorite topic. Different from diagnostic ultrasonography, interventional ultrasonography focuses on target selection and needling techniques. Herein, a successful interventional treatment is always based on an initial correct diagnosis, both of which can readily be done with the use of ultrasonographic imaging [29]. Likewise, ultrasound-guided interventional techniques can be incorporated in advanced courses for physicians who already have the basic knowledge of diagnostic ultrasound.

There are several limitations of our study. First, the Likert scale, which is considered ordinal, was used to represent the perceived confidence and usefulness, but was analyzed as interval or continuous variables. Until now, there is still no consensus regarding the best statistical method to investigate these data. Therefore, we have also given the distribution of participants’ responses in detail. Second, the post-workshop assessment was conducted immediately after the hands-on practical session (less than 36 h). The participants might have recalled what they had responded in the pre-workshop evaluation and thus erroneously estimated the improvement of their confidence after the course. Third, although the confidence increased after the course, it might not indicate that the course could improve the accuracy of making a musculoskeletal diagnosis. Therefore, it is important to conduct a validation test to compare the results of ultrasound examinations performed by trainees on patients with basic pathologies detectable by ultrasound to those conducted by experts. If the training course results in increased confidence but little skills, then the training would be inadequate and needs further modification [30]. However, if the trainees’ diagnoses corresponded to those of the experts (for example 90% of the time), then the training would definitely be of benefit. Fourth, the simple nature of the questions administered to the participants should be acknowledged as well as the lack of use of an educational evaluation theoretic model. The four levels of Kirkpatrick’s evaluation model [31], or equivalent would be incorporated in developing our future course.

## Conclusion

A short period ultrasound workshop incorporating the didactic and hand-practice parts might be useful regarding making a musculoskeletal diagnosis by using ultrasound, based on an increase in post-workshop confidence in MSKUS examinations. Our analysis revealed that the perceived confidence of hip scanning was lower than that of other joints after the course, indicating inadequacy of education in hip sonoanatomy and intensity of hands-on practice in the present training program. An increase in the faculty-to-student ratio or length of practice in the hip section should be considered in the future course.

## Additional files

Additional file 1: Pre-Workshop Evaluation. (DOCX 20 kb)

Additional file 2: Post-Workshop Evaluation (DOCX 24 kb)

## Data Availability

The datasets used and/or analyzed during the current study are available from the corresponding author on reasonable request: contact Dr. Ke-Vin Chang (kvchang011@gmail.com).
