# Supplementary material for: Musculoskeletal ultrasound workshops in postgraduate physician training: a pre- and post-workshop survey of 156 participants
Source: BMC Med Educ. 2019 Sep 23;19:362. doi: 10.1186/s12909-019-1769-6 (PMC6755693; doi:10.1186/s12909-019-1769-6)
Supplement: Supplementary file 1 — Pre-Workshop Evaluation. (DOCX 20 kb) [file 12909_2019_1769_MOESM1_ESM.docx]

| **Pre-Workshop Evaluation** | | | | | |
| --- | --- | --- | --- | --- | --- |
| **Name:** | | **Email:** | |  | |
| **Date:** | | **Age:** | | **Gender:** | |
| **Workplace:** ( ).Teaching Hospital ( ). Local Hospital ( ). Private Clinic  ( ).Other________________________________________ | | | | | |
| **Overall work experience in years:** ____________years | | | | | |
| **Specialty:** ( ) General Practitioner; ( ) Neurology; ( ) Orthopedics; ( )Physiatrist; ( )Rheumatology; ( )Other_________________________ | | | | | |
| **Years of experience in performing musculoskeletal ultrasound:** _________ years | | | | | |
| **Access for an ultrasound machine in your workplace:**  ( ) Easy; ( ) Not Easy but Available; ( ) No Machine Available | | | | | |
| **Have you attended similar training courses before?**  **Yes ( ) No ( )** | | | | | |
| If you answer “Yes”, how many similar courses have you attended? **______________**  **Before the workshop, please grade the level of confidence on the background knowledge and skills regarding the following topic** | | | | | |
|  | Not at all | Very little | Some | Confident | Very Confident |
| 1. Sonography of Shoulder Joints | | | | | |
|  | ( ) | ( ) | ( ) | ( ) | ( ) |
| 2. Sonography of Elbow Joints | | | | | |
|  | ( ) | ( ) | ( ) | ( ) | ( ) |
| 3. Sonography of Wrist/Hand Joints | | | | | |
|  | ( ) | ( ) | ( ) | ( ) | ( ) |
| 4. Sonography of Hip Joints | | | | | |
|  | ( ) | ( ) | ( ) | ( ) | ( ) |
| 5. Sonography of Knee Joints | | | | | |
|  | ( ) | ( ) | ( ) | ( ) | ( ) |
| 6. Sonography of Ankle Joints | | | | | |
|  | ( ) | ( ) | ( ) | ( ) | ( ) |
