# Supplementary material for: Musculoskeletal ultrasound workshops in postgraduate physician training: a pre- and post-workshop survey of 156 participants
Source: BMC Med Educ. 2019 Sep 23;19:362. doi: 10.1186/s12909-019-1769-6 (PMC6755693; doi:10.1186/s12909-019-1769-6)
Supplement: Supplementary file 2 — Post-Workshop Evaluation (DOCX 24 kb) [file 12909_2019_1769_MOESM2_ESM.docx]

| **Post-Workshop Evaluation** | | | | | |
| --- | --- | --- | --- | --- | --- |
| **Name:** | | **Date:** | |  | |
| **(1). Please grade the level of confidence regarding the following topics.**  If you think that the topic was more difficult than you expected, it is fine to grade lower confidence than the pre-workshop survey. | | | | | |
|  | Not at all | Very little | Some | Confident | Very Confident |
| 1. Sonography of Shoulder Joints | | | | | |
|  | ( ) | ( ) | ( ) | ( ) | ( ) |
| 2. Sonography of Normal Elbow Joints | | | | | |
|  | ( ) | ( ) | ( ) | ( ) | ( ) |
| 3. Sonography of Normal Wrist/Hand Joints | | | | | |
|  | ( ) | ( ) | ( ) | ( ) | ( ) |
| 4. Sonography of Normal Hip Joints | | | | | |
|  | ( ) | ( ) | ( ) | ( ) | ( ) |
| 5. Sonography of Normal Knee Joints | | | | | |
|  | ( ) | ( ) | ( ) | ( ) | ( ) |
| 6. Sonography of Normal Ankle Joints | | | | | |
|  | ( ) | ( ) | ( ) | ( ) | ( ) |
|  |  |  |  |  |  |
| 1. **Please grade the level of “usefulness” regarding the following topics.** | | | | | |
|  | Not at all | Very little | Some | Useful | Very Useful |
| 1. Sonography of Shoulder Joints | | | | | |
| Lecture | ( ) | ( ) | ( ) | ( ) | ( ) |
| Hands-on | ( ) | ( ) | ( ) | ( ) | ( ) |
| 2. Sonography of Elbow Joints | | | | | |
| Lecture | ( ) | ( ) | ( ) | ( ) | ( ) |
| Hands-on | ( ) | ( ) | ( ) | ( ) | ( ) |
| 3. Sonography of Wrist/Hand Joints | | | | | |
| Lecture | ( ) | ( ) | ( ) | ( ) | ( ) |
| Hands-on | ( ) | ( ) | ( ) | ( ) | ( ) |
|  |  |  |  |  |  |
|  | Not at all | Very little | Some | Useful | Very Useful |
| 4. Sonography of Hip Joints | | | | | |
| Lecture | ( ) | ( ) | ( ) | ( ) | ( ) |
| Hands-on | ( ) | ( ) | ( ) | ( ) | ( ) |
| 5. Sonography of Knee Joints | | | | | |
| Lecture | ( ) | ( ) | ( ) | ( ) | ( ) |
| Hands-on | ( ) | ( ) | ( ) | ( ) | ( ) |
| 6. Sonography of Ankle Joints | | | | | |
| Lecture | ( ) | ( ) | ( ) | ( ) | ( ) |
| Hands-on | ( ) | ( ) | ( ) | ( ) | ( ) |
|  | | | | | |
| **(3) For an advanced ultrasound workshop, which topics would you be “most interested” in? Please choose from the followings (multiple selections are allowed).** | | | | | |
| ( ). Interventional Ultrasound (Ultrasound guided techniques based on joints and different musculoskeletal conditions)  ( ). Ultrasound Imaging for Peripheral Nerve/Muscle System (focusing more on imaging of the peripheral nerves in upper and lower extremities)    ( ). Ultrasound Imaging for the Spine | | | | | |
